# CCAAT-Enhancer Binding Protein-β Expression and Elevation in Alzheimer’s Disease and Microglial Cell Cultures

**DOI:** 10.1371/journal.pone.0086617

**Published:** 2014-01-22

**Authors:** Ron Strohmeyer, Jadd Shelton, Christopher Lougheed, Trisia Breitkopf

**Affiliations:** Department of Biology, Northwest Nazarene University, Nampa, Idaho, United States of America; Harvard Medical School, United States of America

## Abstract

CCAAT-enhancer binding proteins are transcription factors that help to regulate a wide range of inflammatory mediators, as well as several key elements of energy metabolism. Because C/EBPs are expressed by rodent astrocytes and microglia, and because they are induced by pro-inflammatory cytokines that are chronically upregulated in the Alzheimer’s disease (AD) cortex, we have investigated whether C/EBPs are expressed and upregulated in the AD cortex. Here, we demonstrate for the first time that C/EBPβ can be detected by Western blots in AD and nondemented elderly (ND) cortex, and that it is significantly increased in AD cortical samples. In situ, C/EBPβ localizes immunohistochemically to microglia. In microglia cultured from rapid autopsies of elderly patient’s brains and in the BV-2 murine microglia cell line, we have shown that C/EBPβ can be upregulated by C/EBP-inducing cytokines or lipopolysaccharide and exhibits nuclear translocation possibly indicating functional activity. Given the known co-regulatory role of C/EBPs in pivotal inflammatory mechanisms, many of which are present in AD, we propose that upregulation of C/EBPs in the AD brain could be an important orchestrator of pathogenic changes.

## Introduction

Chronic, micro-localized inflammation, consisting primarily of innate and acute phase mechanisms, is widely regarded as a potentially important pathogenic element in Alzheimer’s disease (AD). Nonetheless, despite meticulous cataloging of inflammatory components that are increased in the AD brain (reviewed in [1]), the molecular mechanisms that might underlie or orchestrate such processes are still the objects of intense investigation.

Transcription factors represent a primary point for regulation of gene and subsequent protein expression, and they typically act on sets of genes within multiple pathways. As such, they are in a position to broadly organize cellular responses, including pathogenic responses. CCAAT-enhancer binding proteins (C/EBPs) consist of a family of six transcription factor isoforms that include C/EBPα, C/EBPβ, C/EBPδ, C/EBPε, C/EBPγ, and C/EBPζ (reviewed in [2]–[7]). C/EBPs function as pleiotropic transactivators of a wide array of genes involved in energy metabolism, cell differentiation, and inflammation, most often in concert with other transcription factors [6]. For example, C/EBP family members C/EBPβ and C/EBPδ can be important co-regulators with nuclear factor-κB (NF-κB) in a host of inflammatory responses [8]–[12], although this role appears to have been largely overlooked in AD studies of NF-κB-related mechanisms. Because the protein products of many of the inflammatory genes of which the C/EBPs have been shown to regulate in the periphery [5], [6], [13] are also known to be altered in AD [1], our goal is determining which C/EBP isoforms are expressed in human brain and which are upregulated in pathologically-vulnerable regions of the AD brain.

In a previous study, we reported for the first time that C/EBPδ is present in the human brain and is upregulated in the AD cortex, with predominant localization to astrocytes [14]. Here, we show for the first time that the isoform C/EBPβ is also present in the human brain with increased expression in AD brain microglia by immunohistochemistry and western blot and that C/EBPβ is also expressed and upregulated in the AD brain and in microglial cultures. Further, C/EBP upregulation and nuclear localization in response to lipopolysaccharide (LPS) and pro-inflammatory cytokines is also shown in primary human elderly microglia cell cultures and BV-2 murine microglia cell line cultures. These results are consistent with recent findings showing a key role for C/EBPβ in regulating microglial activation in pro-inflammatory environments within the brain [15]–[20].

## Materials and Methods

### Ethics Statement

Northwest Nazarene University is committed to the highest standard of integrity in research. All human research activities are governed by the principles outlined in Title 45 Code of Federal Regulations Part 46.

The University’s Code of Conduct for the Responsible Practice of Research sets out the obligations on all University researchers, staff, and students to be aware of the ethical framework governing research at the University and to comply with institutional and regulatory requirements.

All human tissues and cells used in this study meet the criteria for NIH exemption 4 for human research as determined by the Banner Sun Health Research Institute Brain Bank and per approval by the Northwest Nazarene University Human Research Review Committee and by the University of Idaho and the Idaho INBRE grant IRB approval.

### 2.1 Brain Autopsy Specimens

All brain tissues for both glial cell cultures (2.2) and immunohistochemistry (2.3) were kindly provided by the Banner Sun Health Research Institute Brain Bank. (http://www.bannerhealth.com/Research/ResearchInstitutes/BannerSunHealthResearchInstitute/Research/ResearchPrograms/BrainandBodyDonation/Tissuerequest.htm). Neocortex and limbic cortex samples were dissected from routine autopsies of AD and ND patients. Postmortem intervals averaged less than 3 hours, and did not exceed 4 hours. Complete neuropathologic evaluations were performed in all cases. AD and ND groups were well-matched for age, gender, and postmortem interval, and did not differ significantly with respect to these variables (data not shown) [21].

### 2.2 Elderly Microglia Cultures

The method for developing isolated microglia from rapid autopsies of AD and ND patients, including a relatively complete characterization of these cells, has been previously published [22]–[26]. Microglia cultures have been characterized with respect to their morphology, immunoreactivity, repertoire of inflammation-related secretory activity, and chemotactic and phagocytic responsiveness to amyloid β peptide (Aβ) [23], [24], [26], [27]. They are non-immunoreactive for multiple neuronal, astrocyte, oligodendrocyte, or fibroblast markers, but are immunoreactive for multiple microglial markers [23]. They secrete a wide range of inflammatory proteins, including C1q, interleukin-1 (IL-1), IL-6, IL-8, tumor necrosis factor-α (TNF-α), macrophage colony stimulating factor (M-CSF), monocyte chemoattractant protein-1 (MCP-1), and macrophage inflammatory protein-1α (MIP-1α), and increase such secretion after exposure to amyloid β peptide (Aβ) [24]. They express the receptor for advanced glycation endproducts (RAGE) [27] and the formyl peptide receptor [28]. They exhibit pronounced chemotaxis to and phagocytosis of Aβ [25], [26]. For the present experiments, isolated microglia cultures were used two weeks post-plating, a time point when the cells appear to be highly stable (e.g., firm attachment, process elaboration), optimally viable (e.g., by trypan blue exclusion), and phenotypically and immunotypically homogenous at approximately 95% or more purity [23]. Prior to specific experiments, randomly selected sample wells were sacrificed for characterization so as to insure viability and specificity. For viability, cell counts were performed on 0.3% trypan blue stained cultures using a simple hemocytometer. Unstained viable cells and stained dead cells were counted within 3–5 min of dye exposure (data not shown).

### 2.3 Immunohistochemistry and Immunocytochemistry

Neocortex and limbic cortex dissections for immunohistochemistry were post-fixed in 4% buffered paraformaldehyde for 24–36 hours, cryoprotected, sectioned at 40 µm on a freezing microtome, and stored at −20°C in 33% glycerin, 33% glycerol, 33% phosphate buffer (pH 7.4) solutions. After blocking in 3% bovine serum albumin, the sections were incubated overnight in rabbit polyclonal C/EBPβ antisera (Active Motif, Carlsbad, CA–no longer available) (0.785 µg/ml) or C/EBPβ antisera Δ198 1:500 (Santa Cruz Biotechnology Inc., Santa Cruz, CA). Detection of immunoreactivity employed Vectastain kits (Vector Laboratories, Burlingame, CA) and chromogenic detection with diaminobenzidine (DAB). Selected sections were also double-labeled with antibodies to the astrocyte marker GFAP (1∶5000) (Sternberger Monoclonals, Lutherville, MD), antibodies to the activated microglia marker LN3 (1∶1000) (HLA-DR–MHC Class II) (ICN Pharmaceuticals, Costa Mesa, CA), or antibodies to Aβ17–24 (4G8) (1∶1000) (Signet, Dedham, MA). Secondary antibodies were species-specific Alexa Dye-conjugated antibodies (Molecular Probes, Eugene, OR). Image analysis of percent C/EBPβ staining was done using ImageJ (Rasband, W.S., ImageJ, U. S. National Institutes of Health, Bethesda, Maryland, USA, http://imagej.nih.gov/ij/, 1997–2012).

For in vitro immunocytochemistry, microglia were plated at a density of 20,000 cells/well in four-well slide chambers (Fisher-Nunc, Pittsburgh, PA). After two days the cultures were switched to serum-free Dulbecco’s Modified Essential Medium (DMEM – Hyclone) for 12 hours, then exposed to 50 ng/ml IL-1β, IL-6, TNF-α (R&D Research, Minneapolis, MN), or vehicle for 4 hours, washed 3X for 5 min each in cold phosphate buffered saline (PBS), fixed 15 min in cold 4% buffered paraformaldehyde, washed 3X for 5 min each in cold PBS, blocked in 3% bovine serum albumin, and incubated overnight with 1 ml of rabbit polyclonal anti-C/EBPβ antisera (Active Motif - no longer available) (0.785 µg/ml). Detection of immunoreactivity employed biotinylated secondary antibodies with Alexa Dye-conjugated streptavidin (Molecular Probes). The cultures were imaged using an Olympus Fluoview confocal microscope. All cultures were imaged on the same day with the same photomultiplier tube intensity, gain, and offset settings so as to permit qualitative comparisons of fluorescence intensity under the various conditions. Image analysis to quantify intensity of immunostaining was done using ImageJ.

### 2.4 Western Blot Analysis

Tissue from neocortex was homogenized in 3 ml radioactive immunoprecipitation assay (RIPA) buffer, 0.1% sodium dodecyl sulfate (SDS), and 1 tablet Complete protease inhibitor cocktail (Boehringer-Mannheim, Indianapolis)/50 ml buffer, maintaining a temperature of 4°C throughout. After 30 min incubation on ice, the samples were centrifuged at 35000×*g* in an ultracentrifuge for 1 hour at 4°C. The supernatant was put into 1.5 ml tubes into a microcentrifuge and spun at full speed (≈14000 *rpm*) for 10 min to clear debris, and stored at −80°C until assay. To each lane, 30 µg protein was loaded into 4–10% gradient gels (Invitrogen, Carlsbad, CA), with electrotransfer onto 0.45 µm nitrocellulose membranes. After blocking with 5% nonfat dry milk in PBS/Tween for 2 hours, the membranes were incubated overnight at 4°C with C/EBPβ antibody (0.314 µg/ml) (Active Motif – no longer available) or C/EBPβ antibody C19 1:500 (Santa Cruz Biotechnology Inc., Santa Cruz, CA). Santa Cruz’s H7 C/EBPβ antibody did not work for western blotting or immunocytochemistry. Following 3 washes in PBS/Tween, horseradish peroxidase-conjugated secondary antibody (Pierce-Thermo Scientific, Rockford, IL) diluted 1∶10,000 in 5% nonfat dry milk/PBS/Tween was added to the blots for 3 hours at room temperature. Finally, the blots were washed with PBS/Tween, developed with the Super Signal West Pico detection system (Pierce, Rockford, IL), and exposed to film. The blots were also reprobed with β-actin antibody (Sigma, St. Louis) at 1∶10,000 as an internal control. Standard scanning densitometry (ChemiImager, Alpha Innotech, San Leandro, CA) was performed on the immunoreactive bands, with normalization of densitometry measures to β-actin.

### 2.5 BV-2 Cell Line Cultures

BV-2 cells are derived from primary mouse microglia cells [29] and have become an acceptable and widely used alternative model for primary microglia, exhibiting virtually the same inflammatory profile as primary microglia [30]. BV-2 cells (kindly provided by Dr. Gary Landreth, Case Western Reserve University, Cleveland, Ohio but are also available from Interlab Cell Line Collection, Banca Biologica e Cell Factory, Italy *BV*-*2 mouse microglia cell line ICLC ATL03001* http://www.iclc.it/listanuova.html) were incubated at 37°C, 5% CO_2_ and 95% relative humidity in Dulbecco’s Modified Eagle Medium (DMEM - Hyclone) supplemented with 10% FBS (Hyclone) and antibiotic (gentamicin 25 µg/ml - Hyclone). Cells were cultured as above but with 2% serum for 24 hrs before treatments. Treatments of BV-2 cells were always performed in serum-free DMEM. Density and viability of the cells was controlled by counting random fields with trypan blue (Sigma). For immunocytochemistry experiments, BV-2 microglia cells were cultured in four-well slide chambers (Fisher-Nunc) in 1 ml of media at a density of 20,000 cells/well.

### 2.6 Cytoplasmic and Nuclear Extracts

For analysis, the 1×10^6^ cells per well of a six-well plate of BV-2 cells were stimulated for varying times up to 24 hours with LPS (1 µg/ml – Sigma), aggregated Aβ1–42 (1 or 5 µM), and/or IL-6 (10 or 50 ng/ml – R&D Research) and then whole cell, cytoplasmic and nuclear protein extracts were prepared using a nuclear extract kit (Active Motif Cat. #40410). Cells are collected by removing culture media and washing with 5 ml ice-cold PBS/Phosphatase Inhibitors and then aspirating solution out and adding 3 ml ice-cold PBS/Phosphatase Inhibitors. Cells are gently scraped and transferred to a pre-chilled 15 ml conical tube and centrifuged for 5 min at 500 rpm in a table top centrifuge pre-cooled at 4°C. Supernatant was discarded and cell pellet resuspended in 500 µl 1X Hypotonic Buffer, transferred to a pre-chilled microcentrifuge tube, and incubated for 15 min on ice. Then, 25 µl detergent was added, vortexed 10 seconds at highest setting, and centrifuged for 30 seconds at ≈14,000 rpm in a microcentrifuge pre-cooled at 4°C. Supernatant from the cytoplasmic fraction was aliquoted into a pre-chilled microcentrifuge tube and stored at –80°C.

The pelleted nuclei remaining were resuspended in 50 µl Complete Lysis Buffer, vortexed 10 seconds at highest setting, and incubated for 30 min on ice on a rocking platform set at 150 rpm. The nuclear suspension was then vortexed 30 seconds at highest setting and centrifuged for 10 min at 14,000 rpm in a microcentrifuge pre-cooled at 4°C. Supernatant (nuclear fraction) was transferred to a pre-chilled microcentrifuge tube and stored at –80°C. Whole cell extracts were prepared by placing collected cells directly into Complete Lysis Buffer.

Protein Quantification was determined using the micro BCA protocol and samples diluted 100X (Pierce-Thermo Fisher Scientific Inc., Rockford, IL).

## Results

### 3.1 C/EBPβ Immunocytochemistry in Primary Microglia from Elderly Human Brains and Murine BV-2 Microglia Cell Line Cells

**Figure 1:**
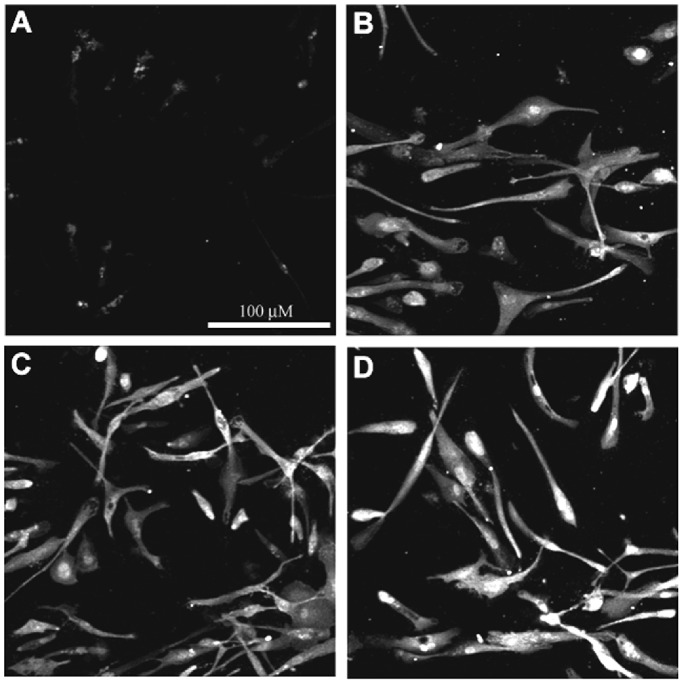
C/EBPβ confocal fluorescent immunocytochemistry in human microglia cultures. Parallel wells received (A) no cytokine treatment or 50 ng/ml (B) IL-1β, (C) IL-6, or (D) TNF-α for 4 hours. Microglia treated with cytokines show increased expression and nuclear localization of C/EBPβ. All wells were imaged concurrently with the same photomultiplier tube intensity, gain, and offset settings using an Olympus Fluoview Confocal microscope–200X (scale bar = 100 µm).

**Figure 2:**
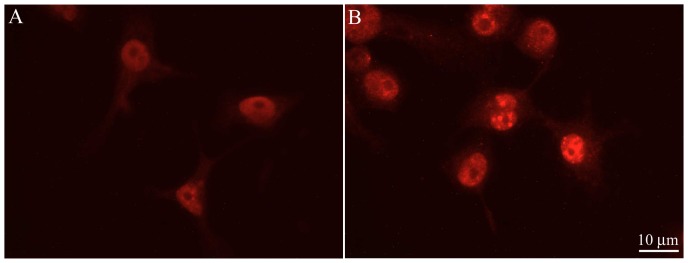
C/EBPβ immunocytochemistry of cultured murine BV-2 cell line. C/EBPβ is increased both in numbers of immunopositive cells and in intensity of fluorescence in the nucleus of cells treated with (B) 1 µg/ml LPS as compared with (A) controls (scale bar = 10 µm). Some control cells were positive for C/EBPβ but the immunofluorescence was notably less intense. The average mean gray value of pixels in LPS treated cells (51.14±3.15, SEM) was significantly higher than the control (38.45±1.86, SEM) (P<0.05). Integrated density in LPS treated cells (6.87±0.42, SEM) was also significantly higher than the control (4.23±0.31, SEM) (P<0.01).

Cell cultures were employed to determine whether or not human microglial expression of C/EBPβ is associated with functionally significant properties such as C/EBPβ responsiveness to cytokines by upregulation and nuclear localization. Under the confocal conditions used throughout, C/EBPβ immunoreactivity after vehicle only exposure was difficult to detect (Figure 1A), although it could be clearly visualized after digital-enhancement (not shown). Treatment with 50 ng/ml of the C/EBP-inducing pro-inflammatory cytokines IL-1β, IL-6, or TNF-α for 4 hours (Figure 1B-1D, respectively) dramatically increased C/EBPβ immunofluorescence in microglia. Nuclear immunoreactivity was also evident after cytokine stimulation, consistent with functional translocation of the transcription factor to the nucleus (Figure 1B–1D). A similar increase in C/EBPβ expression with nuclear localization was observed in the murine BV-2 cell line cells [30] treated with LPS (Figure 2B) as compared with control cells (Figure 2A). Image analysis was done to quantitatively determine differences in staining intensity between control and LPS treated cells. The average mean gray value of pixels in LPS treated cells (51.14±3.15, SEM) was significantly higher than the control (38.45±1.86, SEM) (P<0.05). Similarly, the integrated density in LPS treated cells (6.87±0.42, SEM) was significantly higher than the control (4.23±0.31, SEM) (P<0.01). Integrated Density is the sum of the values of the pixels in the image or selection, which is equivalent to Area × Mean Gray Value.

### 3.2 Nuclear Localization of C/EBPβ in BV-2 Cells Observed by Western Blot

**Figure 3:**
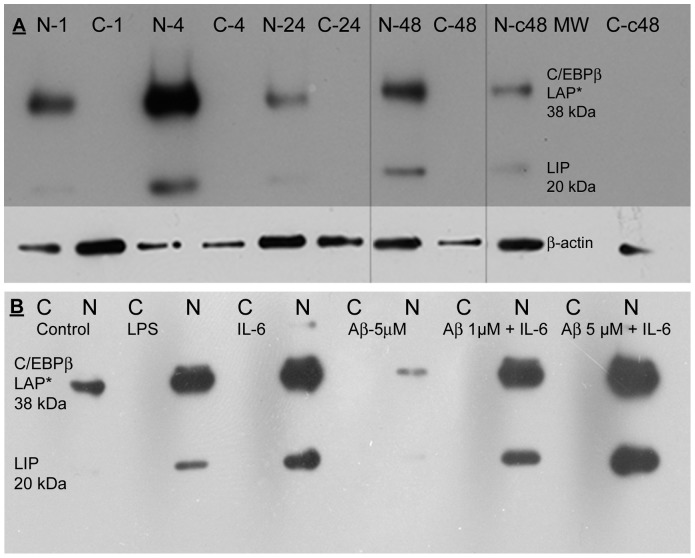
C/EBPβ in treated BV-2 cell Western blots. (A) BV-2 cells treated with 1 µg/ml LPS for differing times. Maximal expression of C/EBPβ (including the LIP isoform) is induced in nuclear fractions (NF) at 4 hours relative to 1, 24, or 48 hours, or 48 hour untreated controls. Cytoplasmic fractions (CF) show little to no expression of C/EBPβ. Lanes: (1) 1 hour NF (2) 1 hour CF (3) 4 hour NF (4) 4 hour CF (5) 24 hour NF (6) 24 hour CF (7) 48 hour NF (8) 48 hour CF (9) 48 hour Control NF (10) molecular weight marker–not visible (11) 48 hour Control CF (12) Blank. β-actin control shown in lower panel. (B) BV-2 cells treated with 1 µg/ml LPS or 10 ng/ml IL-6 and/or 1 or 5 µM aggregated Aβ(1–42). C/EBPβ expression was increased in cells treated with LPS and further increased by IL-6 treatment. Odd numbered lanes are corresponding cytoplasmic fractions for nuclear fractions (NF). Even numbered lanes are nuclear fractions: (2) Control untreated (4) LPS (6) IL-6 (8) Aβ 5 µM (10) Aβ 1 µM+IL-6 (12) Aβ 5 µM+IL-6.

BV-2 microglia cells were cultured at 1×10^6^ cells/well in six-well plates and treated with 1 µg/ml LPS, 10 ng/ml IL-6 and/or 1 or 5 µM aggregated Aβ(1–42). LPS and IL-6 are both potent activators of C/EBPβ expression and activation. Whole cell, nuclear, and cytoplasmic extracts were prepared from treated cells, protein concentration determined, and subjected to SDS-PAGE and Western blotted. Whole-cell extracts were positive for C/EBPβ (not shown). Subsequent analysis showed that cytoplasmic fractions had little to no C/EBPβ expression (Figure 3A and 3B). However, nuclear fractions revealed the clear presence of C/EBPβ proteins that increased in expression over control with LPS (Figure 3A and 3B) or IL-6 treatment (Figure 3B). Expression in the nuclear fraction is consistent with activation of the transcription factor. Time course treatment of BV-2 cells with LPS for 1, 4, 24, and 48 hours showed that C/EBPβ levels peak at 4 hrs, decrease by 24 hrs and begin to increase again at 48 hrs in nuclear fractions (Figure 3A). BV-2 cells treated for 4 hrs with LPS alone, IL-6 alone, Aβ(1–42) alone, and IL-6+Aβ(1–42) show a marked increase of C/EBPβ in LPS and IL-6 treated samples as compared with Aβ(1–42) treated samples and control untreated (Figure 3B). These results show that C/EBPβ expression is induced in BV-2 microglia cells by the inflammatory mediators IL-6 and LPS, consistent with its known regulation by IL-6 and induction by LPS [6], [31]. Cells treated with Aβ(1–42) alone are at or below control levels. Cells treated with both IL-6 and Aβ(1–42) show increased levels but this is likely primarily due to the IL-6. It was initially expected that Aβ(1–42) would cause an increased expression of C/EBPβ in BV-2 microglial cells. However, our results may be due to using highly aggregated Aβ(1–42), which may not effectively activate BV-2 microglial cells and/or due to the manner in which it was prepared [32]–[34]. All treatments also revealed a marked expression of the liver-enriched inhibitory protein (LIP) isoform of C/EBPβ, consistent with observed LIP upregulation by LPS [31].

### 3.3 C/EBPβ Western Blots in AD and ND Cortical Samples

**Figure 4:**
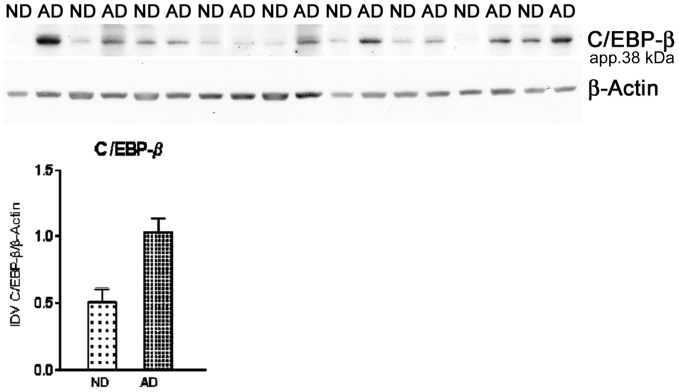
C/EBPβ Western blots in AD and ND frontal lobe gray matter. When normalized to β-actin, the data show a significant increase in C/EBPβ protein expression in AD compared to ND samples (t_16_ = 2.9, P<0.01). Summary densitometry data for the blots (mean ratios of C/EBPβ/β-actin integrated optical density values, IDV) are provided in the bottom panel.

Expression of C/EBPβ in human brain tissues was investigated using two experimental approaches, western blotting and immunohistochemistry. Western blots both confirmed the presence of C/EBPβ and provided a quantitative assessment of C/EBPβ levels for comparison of ND and AD brains. Western blots for C/EBPβ in AD and ND homogenates prepared from frontal lobe gray matter revealed a major immunoreactive band at approximately 38–41 kD (Figure 4), consistent with the 38 kD band reported by others [35]. Densitometry revealed a significant elevation of C/EBPβ (t_16_ = 2.9, P<0.01) immunoreactivity in AD as compared to ND cortical samples (Figure 4).

### 3.4 C/EBPβ Immunohistochemistry in AD and ND Tissue Sections

**Figure 5:**
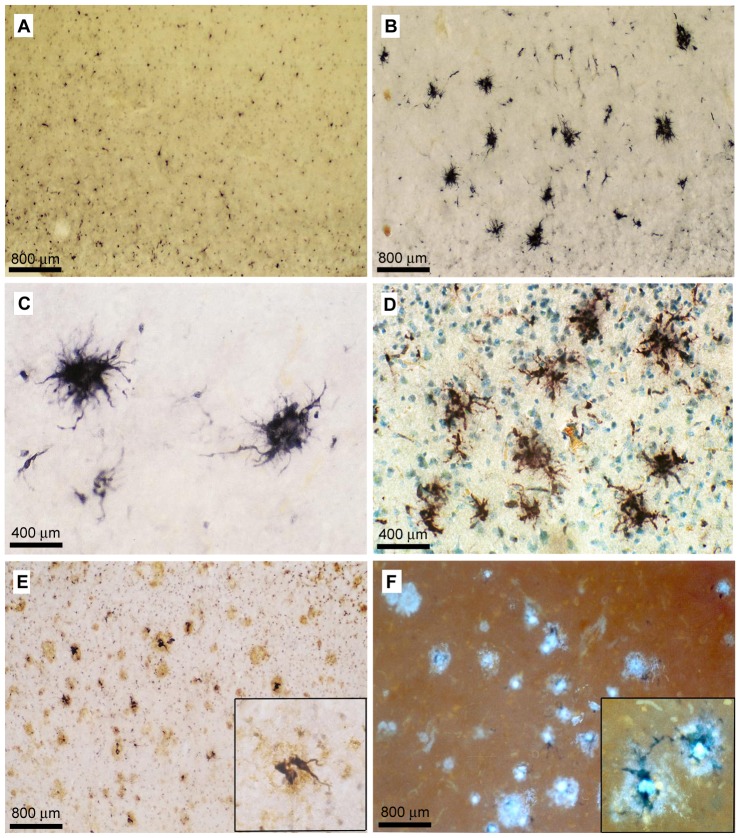
C/EBPβ bright field immunohistochemistry. Single and dual-label immunohistochemistry demonstrates increased immunoreactivity for C/EBPβ on activated AD microglia invested in amyloid plaques in AD tissue as compared to ND tissue. C/EBPβ immunoreactivity in (A) ND SFG tissue, (B and C) AD SFG tissue, and (D) AD entorhinal cortex tissue. (A–B–200X, scale bars = 800 µm. C–D–400X, scale bars = 400 µm). Percent area of C/EBPβ immunostaining is higher in AD (B: 2.23%) vs. ND (A: 0.44%). (E) C/EBPβ immunoreactive cells (black) and 4G8-immunoreactive Aβ plaques (brown) in AD SFG tissue. (F) C/EBPβ immunoreactive cells (black) and thioflavine S-labeled Aβ plaques (green fluorescence) in AD SFG tissue. (E–F–200X, inset 400X). Significantly higher percentages of C/EBPβ immunopositive cells were associated with Aβ plaques (E: 96.9%, F: 89.4%, both Student’s *t*-test, P<0.01) than not associated with Aβ plaques.

Consistent with the Western blot results, C/EBPβ immunoreactivity was markedly increased in AD compared to ND frontal pole brain tissue sections (Figure 5). Image analysis demonstrated a higher percent area of C/EBPβ immunostaining in AD (2.23%) vs. ND (0.44%) tissues (Figure 5B and 5A, respectively). C/EBPβ immunoreactivity localized to microglia (Figures 5A–5D). This was especially true of profusely labeled cells with the morphology and pervasive inundation of Aβ deposits characteristic of microglia in the AD brain (Figures 5E–5F). Quantitation revealed that an average of 96.9% (Figure 5E) and 89.4% (Figure 5F) of C/EBPβ immunostained microglia cells were associated with Aβ plaques, significantly higher than C/EBPβ immunostained microglia cells not associated with plaques (P<0.01). C/EBPβ immunostaining of cells with the morphology or unique peri-plaque localization of astrocytes was not observed, although such astrocyte immunoreactivity for C/EBPδ could be readily demonstrated (c.f., [14]). ND tissue exhibited minimal diffuse staining of microglia (Figure 5A) consistent with reported low levels of microglial activation (as demonstrated by MHC II immunoreactivity) in the elderly cortex [36]–[38]. Primary antibody deletion resulted in a complete abolition of staining (not shown).

**Figure 6:**
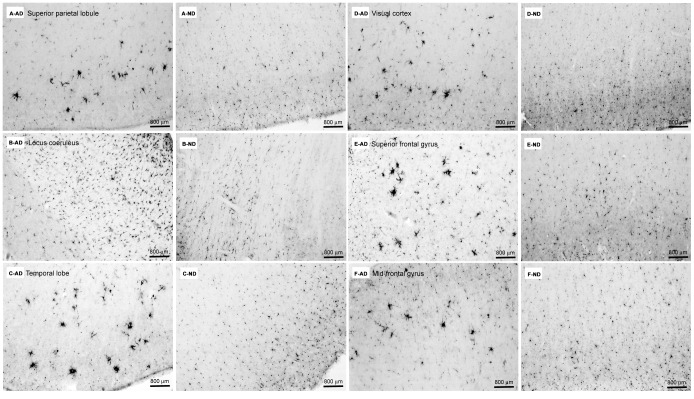
C/EBPβ bright field immunohistochemistry across multiple brain regions. Comparisons of C/EBPβ immuno-labeling across six different brain regions demonstrates increased immunoreactivity for C/EBPβ on activated AD microglia in AD tissue as compared to ND tissue. As shown in Figure 5 and 7, clustered activated microglia in the AD brain are almost invariably associated with Aβ plaques. Percent area of C/EBPβ immunostaining by image analysis is shown in parentheses. (A) Superior parietal lobule (AD 0.54% vs. ND 0.3%, AD/ND ratio = 1.8). (B) Locus coeruleus (AD 2.2% vs. ND 0.29%, AD/ND ratio = 7.6). (C) Temporal lobe (AD 1.18% vs. ND 0.6%, AD/ND ratio = 2). (D) Visual cortex (AD 0.56% vs. ND 0.47%, AD/ND ratio = 1.2). (E) Superior frontal gyrus (AD 1.36% vs. ND 0.47%, AD/ND ratio = 2.9). (F) Mid-frontal gyrus (AD 0.63% vs. ND 0.55%, AD/ND ratio = 1.1). (All micrographs are at 200X, scale bars = 800 µm).

C/EBPβ immunohistochemistry of 6 additional brain regions demonstrated a consistent pattern of microglia staining in AD, particularly microglia investing amyloid plaques, in brain regions impacted by AD (Figures 6A–6F–AD). Image analysis consistently showed a higher percent area of C/EBPβ positive staining in AD vs. ND tissues and the ratio of AD/ND percent area immunostained was greater than 1 for all regions (Figure 6). Superior parietal lobule (AD 0.54% vs. ND 0.3%, AD/ND ratio = 1.8); Locus coeruleus (AD 2.2% vs. ND 0.29%, AD/ND ratio = 7.6); Temporal lobe (AD 1.18% vs. ND 0.6%, AD/ND ratio = 2); Visual cortex (AD 0.56% vs. ND 0.47%, AD/ND ratio = 1.2); Superior frontal gyrus (AD 1.36% vs. ND 0.47%, AD/ND ratio = 2.9); Mid-frontal gyrus (AD 0.63% vs. ND 0.55%, AD/ND ratio = 1.1) Cerebellum, a brain region largely spared in AD, demonstrated very little immunoreactivity for C/EBPβ (not shown). As with the frontal pole sections, ND tissue from these additional brain regions exhibited minimal diffuse staining of microglia that varied in degree of intensity by brain region (Figures 6A–6F–ND). Again, this is consistent with reported low levels of microglial activation in the nondemented elderly cortex [36]–[38].

### 3.5 Colocalization of C/EBPβ Immunoreactivity by Confocal Microscopy in AD Tissue Sections with Microglial and Aβ Markers

**Figure 7:**
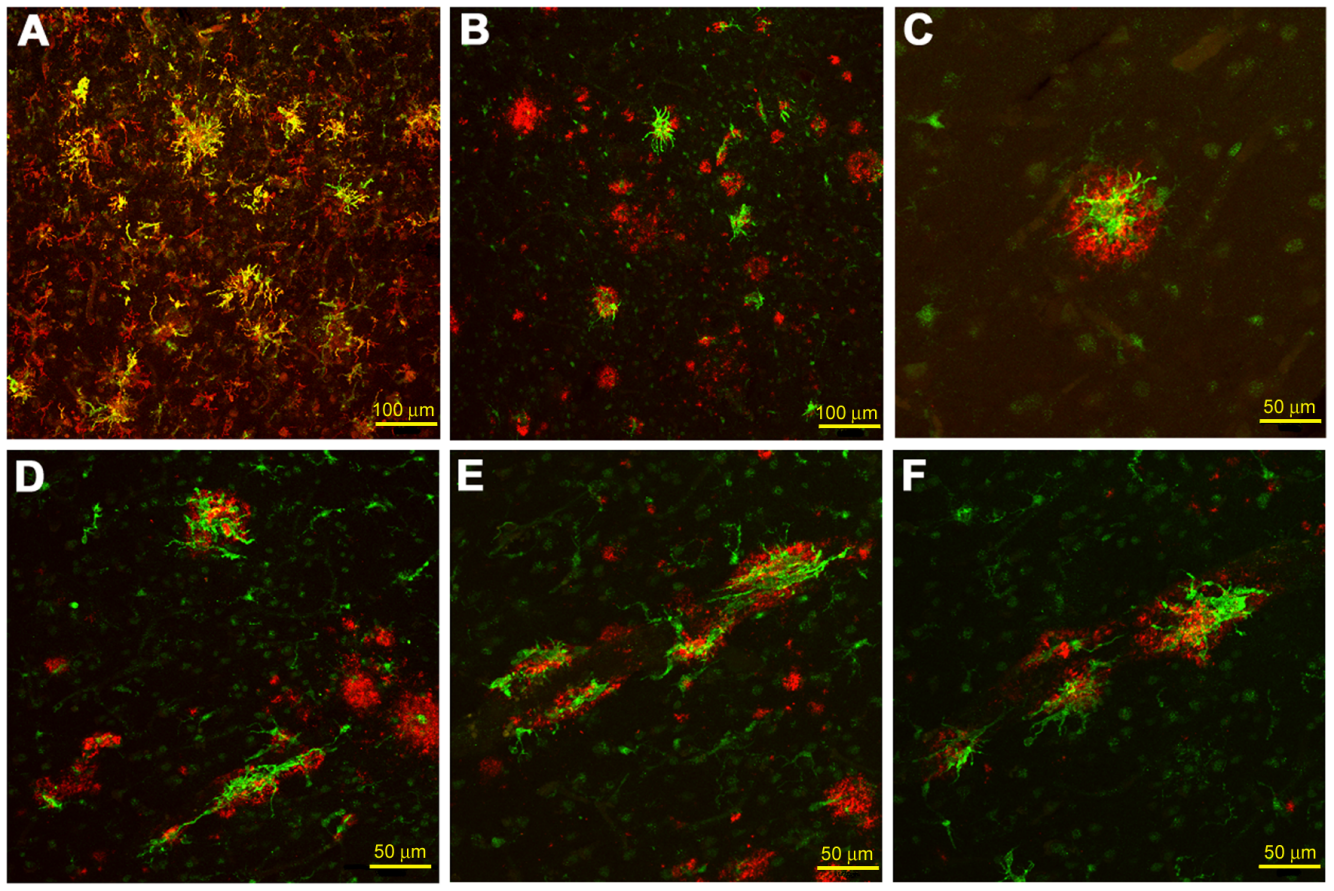
C/EBPβ confocal fluorescent immunohistochemistry of AD superior frontal gyrus brain tissue. (A) C/EBPβ positive cells (green) and HLA-DR positive activated microglia (red) (MHC Class II–activated microglial marker) colocalize together (yellow), positively identifying C/EBPβ immunoreactive cells as activated microglia–200X (scale bar = 100 µm), with 55% of microglia being C/EBPβ immunopositive. (B–D) C/EBPβ positive activated microglia (green) colocalize with 4G8-immunoreactive Aβ plaques (red)–200X and 400X (scale bar = 50 µm). (D–F) C/EBPβ positive activated microglia (green) colocalize with 4G8-immunoreactive Aβ-laden microvasculature (red)–400X. Percentages of C/EBPβ immunopositive cells associated with Aβ: (B) 72.4%, P<0.01 (D) 55.4%, (E) 59.1%, (F) 48.1%. Upon averaging, the number of C/EBPβ immunopositive cells associated with Aβ was significantly higher than C/EBPβ immunopositive cells not associated with Aβ (Student’s *t*-test, P<0.01).

To confirm that the immunoreactive cells were indeed microglia, and to demonstrate that C/EBPβ immunoreactive microglia were highly associated with Aβ plaques in the AD brain, sections from the superior frontal gyrus (SFG) of AD patients were double-labeled with microglia (LN3) and Aβ-specific (4G8 and 6E10) antibodies or thioflavine S. These sections also revealed that C/EBPβ immunoreactivity colocalized with microglia investing Aβ plaques and, again, failed to detect C/EBPβ-positive astrocytes (by GFAP antibody-not shown). Confocal microscopy showed colocalization of C/EBPβ immunoreactivity with immunoreactivity for the conventional microglial activation marker HLA-DR (MHC Class II) (LN3 monoclonal antibody), with quantitative image analysis indicating that 55% of LN-3-labeled microglia were C/EBPβ-positive. (Figure 7A). Confocal microscopy also revealed colocalization of C/EBPβ immunoreactive microglia with Aβ in both amyloid plaques (Figures 7B–7D) and amyloid-laden microvasculature (Figures 7D–7F). Upon quantitation, percentages of C/EBPβ positive cells associated with Aβ were as follows: (7B) 72.4%, P<0.01 (7D) 55.4%, (7E) 59.1%, (7F) 48.1%. When values were averaged together, C/EBPβ positive cells associated with Aβ were significantly higher than C/EBPβ-positive cells not associated with Aβ (P<0.01).

## Discussion

We have previously reported that C/EBPδ is present in the human brain, upregulated in AD brain astrocytes, and functionally responsive to cytokine stimulation in human post-mortem astrocyte cell cultures [14]. Here, we extend our study of C/EBP transcription factors by demonstrating that C/EBPβ is also present in the human brain, but is expressed primarily by activated microglia. Particularly strong colocalization of C/EBPβ immunoreactivity with Aβ deposits and Aβ immunoreactive microvessels, profuse immunoreactivity in pathologically-vulnerable regions of the AD brain compared to relatively weak staining in those same regions of ND brain or pathologically-spared regions of the AD brain, and significant C/EBPβ increases in AD cortex compared to ND cortex by Western blot analysis collectively suggest that upregulation of C/EPBβ in the AD brain may be pathophysiologically relevant. In addition, like C/EBPδ [14], C/EBPβ exhibits induction and translocation to the nucleus in cell culture models after exposure to known C/EBP-inducing cytokines or lipopolysaccharide, suggesting that C/EBPβ may be functionally active.

Although in tissue sections C/EBPδ appears to be highly localized to astrocytes (c.f., [14]) and C/EBPβ to microglia (Figures 1, 2 and 5–7), it should be noted that in subsequent studies we have been able to detect C/EBPβ induction and nuclear translocation in astrocyte cultures after stimulation with C/EBP-inducing cytokines (not shown). The basis for this discrepancy with our in situ findings is not clear, but likely reflects innate differences in in vitro and in situ conditions (culture artifact perhaps), particularly with respect to states of activation, accessibility of stimulating factors to receptors, and accessibility of antibodies to their targets.

There are multiple potential mechanisms for the upregulation of C/EBPβ and C/EBPδ in the AD brain, many of which have resonance with known molecular changes in AD. The first level of regulation for C/EBPβ appears to be at transcription, where a number of activators–particularly the pro-inflammatory cytokines IL-1, IL-6, and TNF-α–are known to trigger signaling cascades leading to the mobilization of C/EBPs (autoregulatory mechanism) [6]. This often occurs in concert with other transcription factors such as CREB, STAT-3, and NF-κB, which alone or in combination with each other direct an upregulation in C/EBP mRNA transcription and concomitant protein expression [6]. Indeed, it has been proposed that NF-κB and STAT-3 are early response genes for inflammation that induce the expression of C/EBPβ and C/EBPδ as late response genes in order to sustain the inflammatory response. Autoregulatory mechanisms for C/EBPs then allow for sustained expression of C/EBPβ and C/EBPδ even after NF-κB and STAT-3 mechanisms subside [5].

Once transcribed, regulation of C/EBP effects can be accomplished through several pathways. For example, at the translational level, C/EBPβ regulation can occur via three AUG initiation codons. Leaky ribosome scanning results in three different-sized C/EBPβ isoforms, LAP1 (38 kDa), LAP2 (35 kDa) and LIP (20 kDa), from the same mRNA transcript. LAP2 and LIP are the major forms produced in cells. Whereas LAP1 and LAP2 contain both the transactivation and bZIP domains, LIP contains only the bZIP domain and therefore can act as a dominant negative inhibitor of C/EBP functions by forming non-functional homo- and heterodimers with the various C/EBP isoforms [6], [35]. Our results in Figure 3 show increased expression of C/EBPβ LIP. The C/EBPβ LAP1/2 to LIP ratio as well as dimerization with other C/EBP isoforms appears to be important for regulating gene expression in some important cellular processes [6]. As already noted, LPS treatment has been observed to increase LIP expression. Some observed roles for LAP/LIP include regulation of cellular responses to endoplasmic reticulum stress [39] and live cell engulfment (autophagy) [40]. Additionally LIP can down-regulate C/EBPα expression, a requirement for inflammation [6]. Doubtless, much remains to be learned about this LIP form of C/EBPβ. Nevertheless, aspects of these processes may have some resonance in AD and perhaps LIP is involved similarly in our LPS-treated BV-2 microglial culture models.

The effects of C/EBP isoforms are also highly regulated post-translationally through phosphorylation of a number of key residues. C/EBPβ is normally a repressed factor due to negative regulatory regions that cover the transactivation domains [41], [42]. Signaling via several pathways results in C/EBP phosphorylation and subsequent transport from the cytoplasm to the nucleus [41], [43]–[52]. Conversely, phosphorylation of other C/EBP residues by other signaling pathways can suppress the activation and DNA-binding potential of C/EBPβ [43], [46], [49], [53], [54].

At the physiologic level, the upregulation of C/EBPs in the AD cortex would be expected to have multiple potential effects, many of which, again, have resonance with known AD pathogenic mechanisms. For example, of the cytokines that have received the most attention in AD, IL-1, IL-6, and TNF-α (reviewed in [1]), all are at least partially regulated by C/EBP isoforms [7], [54]–[58]. Complement is known to be activated in AD [1], [59]–[66], where it may have many pathophysiologic actions from opsonization [59], [63] to lysis of bystander neuritis [61]. Expression of the pivotal complement component C3 is co-regulated by C/EBPs [67]–[69]. C-reactive protein, which can activate the complement system [70]–[72], also has a C/EBP binding site in its promoter [73], [74]. The inflammation-related growth factor M-CSF has a C/EBP binding site in its promoter [75], shows pronounced effects on macrophage proliferation and activity, and has been reported to play a key role in RAGE-mediated microglial responses to Aβ [27]. Apolipoprotein E has been implicated in multiple AD pathophysiologic mechanisms, and is likely to be regulated in part by C/EBP [76]. Cytotoxicity of iNOS mechanisms in the AD brain has been extensively studied [77]–[81]. Upregulation of C/EBP results in increased expression of iNOS via direct transcriptional actions [8], [9]. Myeloperoxidase has a C/EBP promoter site as well [82]. Indeed, C/EBP interactions with amyloid precursor protein (APP) or Aβ themselves may warrant investigation, since C/EBP interactions with serum amyloid A and serum amyloid P production have been reported [12], [73], [83]. In addition to promoting the production of inflammatory mediators such as IL-1, IL-6, and TNF-α, C/EBP isoforms are themselves induced by this same classical pro-inflammatory triad [5], [7], [84]–[86]. This puts C/EBP isoforms in a position to both amplify and orchestrate a host of cytokine-mediated effects.

Recent work from a group in Barcelona, Spain has also demonstrated an important role for C/EBPβ in microglial activation with conditions mimicking neuroinflammation [15]–[19], [87], [88]. They have shown that C/EBPβ expression increases in primary microglia and BV-2 microglia cells with LPS and cytokine treatments and that C/EBP expression is necessary for microglia-mediated neurotoxicity. C/EBPβ also appears to decrease CD200R1 receptor expression by microglia during inflammation, making them less responsive to inhibitory CD200 ligand on neurons [17]. C/EBPβ KO mice in a transient focal cerebral ischemia model showed significantly smaller infarcts, reduced neurological deficits, decreased terminal deoxynucleotidyl transferase-mediated dUTP nick-end labeling-positive cells, decreased intercellular adhesion molecule 1 (ICAM1) immunopositive vessels, decreased extravasated neutrophils and fewer activated microglia/macrophages, compared with their wild-type littermates. Furthermore, GeneChip analysis showed that post ischemic induction of many transcripts known to promote inflammation and neuronal damage was less pronounced [20]. Finally, C/EBPβ is increased in mouse models of amyotrophic lateral sclerosis (ALS) and human spinal cord of ALS patients [15]. Our work supports and extends these results by showing that some of these same processes demonstrated in various culture and animal models and in ALS are occurring in the Alzheimer’s diseased brain. It is likely that investigation of C/EBPs in other neurodegenerative diseases will reveal similar results. Altogether our work here presents a correlational argument linking together known roles for C/EBPβ in inflammation and known components of inflammation in AD and the presence of increased expression of C/EBPβ in AD brain tissues and microglial cells to make a case for further functional studies of C/EBPβ actions in AD and other neurodegenerative diseases.

In summary, a substantial role for C/EBPβ and other C/EBP isoforms in AD is consistent with the milieu of inflammatory mediators and cellular mechanisms present in the AD neocortex and limbic cortex (reviewed in [1]), and could also be a key element in orchestrating and sustaining chronic inflammation there. It is also possible that certain C/EBPs, such as C/EBPβ, may play a key role in neuroinflammation across several neurodegenerative diseases. For these reasons, further research into the functional roles that C/EBPs may be playing in AD and neuroinflammation generally seems warranted.
